# Study of the Genetic Mechanisms of Siberian Stone Pine (*Pinus sibirica* Du Tour) Adaptation to the Climatic and Pest Outbreak Stresses Using Dendrogenomic Approach

**DOI:** 10.3390/ijms252111767

**Published:** 2024-11-01

**Authors:** Serafima V. Novikova, Natalia V. Oreshkova, Vadim V. Sharov, Dmitry A. Kuzmin, Denis A. Demidko, Elvina M. Bisirova, Dina F. Zhirnova, Liliana V. Belokopytova, Elena A. Babushkina, Konstantin V. Krutovsky

**Affiliations:** 1 Laboratory of Genomic Research and Biotechnology, Federal Research Center “Krasnoyarsk Science Center of the Siberian Branch of the Russian Academy of Sciences”, 660036 Krasnoyarsk, Russia; serafima_novikova_11@mail.ru (S.V.N.); oreshkova@ksc.krasn.ru (N.V.O.); vsharov@sfu-kras.ru (V.V.S.); 2 Laboratory of Forest Genomics, Genome Research and Education Center, Institute of Fundamental Biology and Biotechnology, Siberian Federal University, 660041 Krasnoyarsk, Russia; 3 Laboratory of Forest Genetics and Selection, V.N. Sukachev Institute of Forest, Siberian Branch of Russian Academy of Sciences, 660036 Krasnoyarsk, Russia; 4 Department of Genomics and Bioinformatics, Institute of Fundamental Biology and Biotechnology, Siberian Federal University, 660041 Krasnoyarsk, Russia; 5 Krasnoyarsk Regional Center of New Information Technologies, Institute of Space and Information Technologies, Siberian Federal University, 660074 Krasnoyarsk, Russia; dm.kuzmin@gmail.com; 6 Department of High-Performance Computing, Institute of Space and Information Technologies, Siberian Federal University, 660074 Krasnoyarsk, Russia; 7 Laboratory of Forest Protection, M.F. Reshetnev Siberian State University of Science and Technology, 660000 Krasnoyarsk, Russia; sawer_beetle@mail.ru; 8 Laboratory of Forest Zoology, V.N. Sukachev Institute of Forest, Siberian Branch of Russian Academy of Sciences, 660036 Krasnoyarsk, Russia; 9 Laboratory for Monitoring of the Carbon Balance of Terrestrial Ecosystems, Institute of Monitoring of Climatic and Ecological Systems, Siberian Branch of Russian Academy of Sciences, 634055 Tomsk, Russia; bissirovaem@mail.ru; 10 All-Russian Plant Quarantine Center (VNIIKR), Tomsk Branch, 634069 Tomsk, Russia; 11 Khakass Technical Institute, Siberian Federal University, 655017 Abakan, Russia; dina-zhirnova@mail.ru (D.F.Z.); white_lili@mail.ru (L.V.B.); babushkina70@mail.ru (E.A.B.); 12 Institute of Ecology and Geography, Siberian Federal University, 660041 Krasnoyarsk, Russia; 13 Department of Forest Genetics and Forest Tree Breeding, Georg-August University of Goettingen, 37077 Goettingen, Germany; 14 Center for Integrated Breeding Research, George-August University of Goettingen, 37075 Goettingen, Germany; 15 Laboratory of Population Genetics, N. I. Vavilov Institute of General Genetics, Russian Academy of Sciences, 119333 Moscow, Russia; 16 Scientific and Methodological Center, G. F. Morozov Voronezh State University of Forestry and Technologies, 394087 Voronezh, Russia

**Keywords:** ddRADseq, dendrophenotype, drought, environmental stress, genome, genotyping by sequencing, GWAS, *Neodiprion sertifer* Geoff., pine sawfly, SNPs

## Abstract

A joint analysis of dendrochronological and genomic data was performed to identify genetic mechanisms of adaptation and assess the adaptive genetic potential of Siberian stone pine (*Pinus sibirica* Du Tour) populations. The data obtained are necessary for predicting the effect of climate change and mitigating its negative consequences. Presented are the results of an association analysis of the variation of 84,853 genetic markers (single nucleotide polymorphisms—SNPs) obtained by double digest restriction-site associated DNA sequencing (ddRADseq) and 110 individual phenotypic traits, including dendrophenotypes based on the dynamics of tree-ring widths (TRWs) of 234 individual trees in six natural populations of Siberian stone pine, which have a history of extreme climatic stresses (e.g., droughts) and outbreaks of defoliators (e.g., pine sawfly [*Neodiprion sertifer* Geoff.]). The genetic structure of studied populations was relatively weak; samples are poorly differentiated and belong to genetically similar populations. Genotype–dendrophenotype associations were analyzed using three different approaches and corresponding models: General Linear Model (GLM), Bayesian Sparse Linear Mixed Model (BSLMM), and Bayesian-information and Linkage-disequilibrium Iteratively Nested Keyway (BLINK), respectively. Thirty SNPs were detected by at least two different approaches, and two SNPs by all three. In addition, three SNPs associated with mean values of recovery dendrophenotype (Rc) averaged across multiple years of climatic stresses were also found by all three methods. The sequences containing these SNPs were annotated using genome annotation of a very closely related species, whitebark pine (*P. albicaulis* Engelm.). We found that most of the SNPs with supposedly adaptive variation were located in intergenic regions. Three dendrophenotype-associated SNPs were located within the 10 Kbp regions and one in the intron of the genes encoding proteins that play a crucial role in ensuring the integrity of the plant’s genetic information, particularly under environmental stress conditions that can induce DNA damage. In addition, we found a correlation of individual heterozygosity with some dendrophenotypes. Heterosis was observed in most of these statistically significant cases; signs of homeostasis were also detected. Although most of the identified SNPs were not assigned to a particular gene, their high polymorphism and association with adaptive traits likely indicate high adaptive potential that can facilitate adaptation of Siberian stone pine populations to the climatic stresses and climate change.

## 1. Introduction

Requirements for forest ecosystems as a source of wood, the basis of terrestrial biodiversity, the main contributor to carbon sequestration, etc. are constantly increasing. Therefore, it is necessary to optimize the use of forest genetic resources with a priority to their conservation and sustainable reproduction, especially in the context of global climate change with more frequent and prolonged droughts and pest outbreaks [1,2]. This requires a more complete understanding of the genetic mechanisms of adaptation and productivity of forest populations under stress conditions [3]. One of the most effective modern approaches to studying the genetic mechanisms of adaptation and the adaptive potential of forest populations is dendrogenomics [4]. It is a new interdisciplinary approach that allows joint analysis of dendrochronological, dendroclimatological, and genomic data. It opens up new ways to study the temporal dynamics of forest treelines, delineate spatial and temporal population structures, decipher individual tree responses to abiotic and biotic stresses, and evaluate the adaptive genetic potential of forest tree populations [4,5]. These data are needed for the prediction of climate change effects and mitigation of the negative effects, especially in boreal and mountainous forests, such as Western Sayan and West Siberian Plane in Siberia (Russia), where rapid rates of regional temperature rise, significantly exceeding global trends, are multiplied by local warming [6,7].

The significant rate of current change in climate parameters, which is not typical for natural cycles, may not allow species and ecosystems to adapt to such rapid climate changes [8]. A shift in the distribution range due to ongoing climatic events is possible both at its upper and lower boundaries, but different factors will act as triggers [9]. Near the lower limit of growth of a species in mountain ecosystems, such mechanisms may include lack of moisture (drought), fires, and outbreaks of insect pests [6,10].

Genome-wide association studies (GWAS) have been widely used to search for genetic mechanisms of tree growth and adaptation to environmental factors [11,12,13], including also the double digest restriction-site associated DNA sequencing (ddRADseq) approach [14,15] used also in our study. Recent studies have demonstrated that candidate genes for adaptive dendrophenotypes can be effectively mined from GWAS and verified [16].

In the presented here research, to study genetic adaptation to ongoing significant climatic changes in mountain dark coniferous forests, a search was carried out for associations between genetic variation and individual dendrophenotypes calculated on the basis of the dynamics of the tree-ring width (TRW) in Siberian stone pine (*Pinus sibirica* Du Tour) trees in Siberian populations affected by severe climatic extremes and insect defoliator outbreaks, such as of pine sawfly (*Neodiprion sertifer* Geoff.).

## 2. Results

### 2.1. SNP Calling

For 243 Siberian pine samples from six populations, ddRADseq yielded 7.397 billion nucleotide reads with an average length of 141 bp and mean 30.441 million reads per sample. On average, 78.6% of reads from each sample were successfully mapped using bowtie2 to the whitebark pine genome assembly. Nine trees were excluded from further analysis due to insufficient quality of sequencing and mapping. In total, after filtering, 145,402 loci with an average length of 144 bp (standard error, SE = ±0.11 bp) were selected. Among them, 40,553 loci contained 84,853 single nucleotide polymorphisms (SNPs), which were included in the final data set for 234 samples.

### 2.2. Genetic Structure of Populations

For each sample, general parameters of genetic variation, such as nucleotide diversity (π), observed (*H*_o_) and expected (*H*_e_) heterozygosity, and fixation index (*F*_IS_), were calculated and are shown in Table 1.

The pairwise values of the genetic differentiation parameter (*F*_ST_) calculated between populations are presented in Table 2. The maximum *F*_ST_ value was obtained between the TK and PS (0.0188), and the minimum (0.0031)—between the TK and RG populations. The obtained low pairwise *F*_ST_ values between populations indicate that the population samples are relatively poorly differentiated and belong to genetically similar populations.

**Figure 1:**
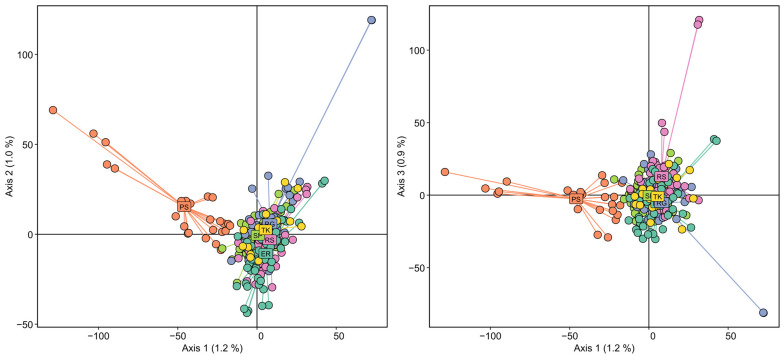
Principal component analysis (PCA) of six populations of Siberian stone pines (PS, TK, ER, RS, RG, and SP) based on allele frequencies.

Principal component analysis (PCA) was performed using allele frequencies to determine population structure. The analysis showed that the studied samples do not form clearly defined unambiguous clusters, but presumably two indicative clusters can be distinguished: one containing trees of the TK, ER, RS, RG, and SP populations, and the other, trees of the geographically distant PS population (Figure 1).

**Figure 2:**
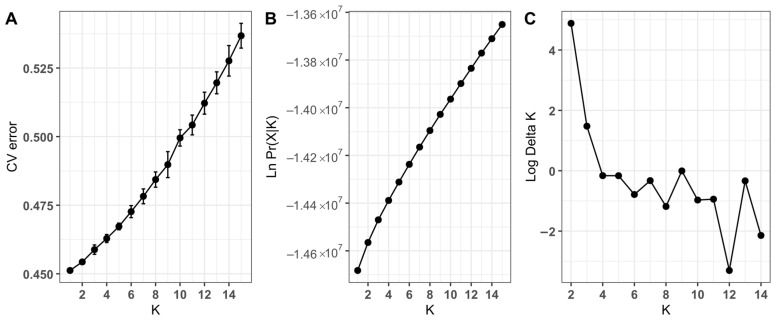
Graphs displaying the results of the analysis of the likely number of clusters *K* in the total sample of Siberian stone pine trees based on the values of CV-error (**A**), log-likelihood probability (**B**), and Δ*K* (**C**).

**Figure 3:**
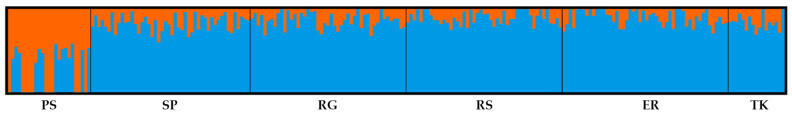
Contribution of each of the clusters (*Q*-values) for *K* = 2 to individual Siberian stone pine trees from six populations (PS, SP, RG, RS, ER and TK).

The Δ*K* method for selecting the most likely number *K* of genetic clusters or subpopulations in the total sample showed that the most likely number was *K* = 2 (Figure 2). The contributions (“admixture”) of each cluster to individual trees (*Q*-values) according to the results of the Admixture program are shown in Figure 3 and Figure S1. Trees of the PS population form a cluster separate from all other populations.

The AMOVA results and estimated Wright fixation indices (*F*-indices) for each level of the population hierarchy, including group level, where the PS population forms a group separate from another group with all other populations (Western Sayan), are presented in Table 3. The highest level of genetic variation was within populations (98.3%), and genetic differentiation between populations was relatively low (*F*_ST_ = 0.017).

**Figure 4:**
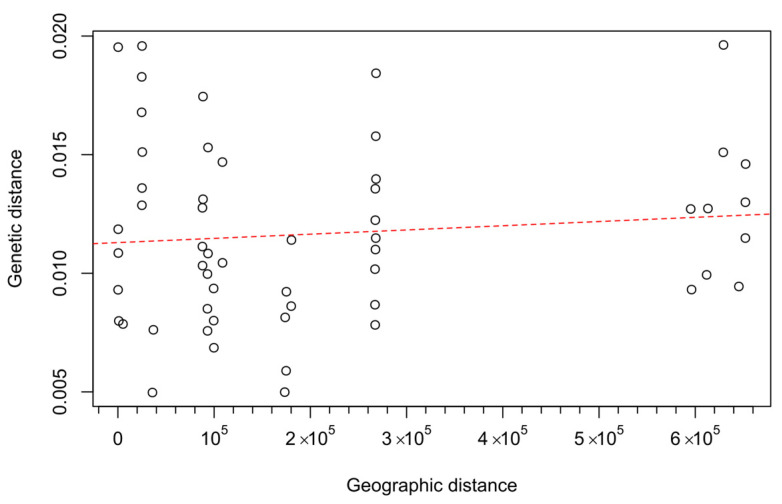
The correlation of pairwise geographic distances with genetic distances based on the Mantel test (*r* = 0.103, *p* = 0.313).

The correlation of pairwise geographic distances with genetic distances was positive but insignificant based on the Mantel test (*r* = 0.103, *p* = 0.313), demonstrating no or weak effect of isolation by distance on genetic differentiation between studied populations (Figure 4).

### 2.3. TRW Growth Trends

**Figure 5:**
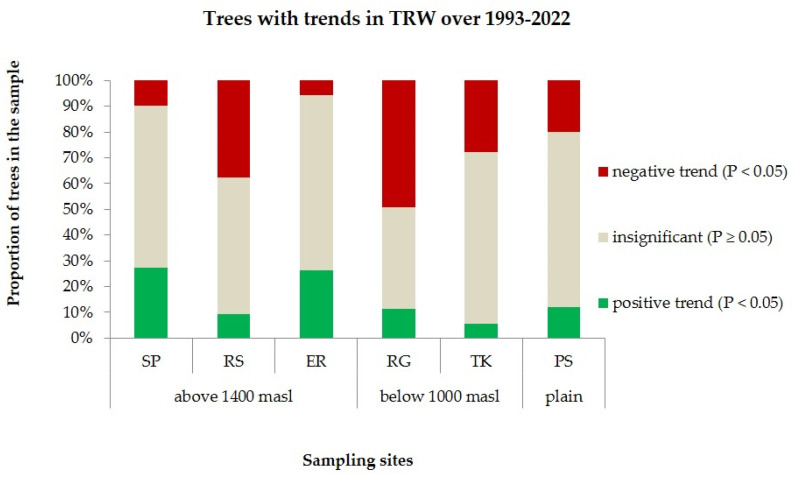
Proportions of trees with positive, negative, and insignificant trends in TRW over last the 30 years of growth sampled in the populations studied in the Western Sayan.

Near the upper forest line, more trees with significant positive trends in growth were observed for the period 1993–2022, while in the low part of the elevational distribution range, more trees with negative trends occurred. The Siberian stone pine population from the West Siberian Plain holds an intermediate position. Nevertheless, 40–68% of trees did not have significant trends across all populations (Figure 5).

### 2.4. Associations Between Dendrophenotypes and the Level of Individual Heterozygosity

For each tree, the individual level of heterozygosity was calculated for all SNPs, averaging 0.212 ± 0.002 (mean ± SE) for all trees. The lowest average values of individual heterozygosity were obtained for the RS population (0.200 ± 0.005) and the highest for the ER population (0.224 ± 0.002). Differences between populations in the level of heterozygosity, according to the Student’s *t*-test (*p* < 0.05), were significant for 9 pairs of populations out of 15: PS-ER (*p* = 0.0001), PS-SP (*p* = 0.0076), TK-ER (*p* = 0.0010), TK-RS (*p* = 0.0061), ER-RG (*p* = 0.0006), ER-RS (*p* = 0.00001), ER-SP (*p* = 0.0338), RG-SP (*p* = 0.0109), RS-SP (*p* = 0.0003). The values of all Pearson and Spearman correlation coefficients between individual heterozygosity and each dendrophenotype, as well as Student’s *t*-test and some other statistics, are presented in Table S1. Based on data for 110 dendrophenotypes, significant correlation values were obtained for only a few dendrophenotypes, but the data are very contradictory. In particular, for the climatic stress resistance indices Rt2, Rt3, and Rt13, the correlations were significantly positive according to both Pearson and Spearman correlation coefficients (Table S1), possibly indicating that more heterozygous trees are also more resistant to growth depression caused by environmental stress factors. However, for Rt1, the correlation was significantly negative according to the Pearson correlation coefficient (*r*_P_ = −0.188, *p* = 0.0362) but positive, although not significant, according to the Spearman correlation coefficient (*r*_S_ = 0.086, *p* = 0.3380). Similarly, for the resilience indices Rs2 and Rs13, the correlations were significantly positive according to both Pearson and Spearman correlation coefficients, but for Rs1, it was significantly negative only according to the Pearson correlation coefficient (*r*_P_ = −0.207, *p* = 0.0204) and non-significant according to the Spearman correlation coefficient (*r*_S_ = −0.009, *p* = 0.9215). At the same time, the resistance and resilience trends coincided for the same periods of environmental extremes.

The highest significant positive correlation between the resistance index (Rt13) during the drought period of 1959–1961 and heterozygosity was observed for trees in the RG population in the inner part of the Siberian stone pine elevational distribution range in the Western Sayan (850–1000 masl) according to both Pearson (*r*_P_ = 0.616, *p* = 0.0006) and Spearman (*r*_S_ = 0.503, *p* = 0.0082) correlation coefficients.

Trees with higher heterozygosity were also more resistant to defoliation caused by pine sawfly in 1972 (defol_1972) in the PS population according to both Pearson (*r*_P_ = 0.444, *p* = 0.0262) and Spearman (*r*_S_ = 0.597, *p* = 0.0016) correlation coefficients.

It is interesting to note that the mean needle length (meanL) and TRW averaged for 30 years (1990–2019) (meanTRW30) calculated for all 234 trees were negatively correlated with the level of individual heterozygosity (*r*_P_ = −0.161, *p* = 0.0137; *r*_S_ = −0.138, *p* = 0.0345; and *r*_P_ = −0.139, *p* = 0.046; *r*_S_ = −0.176, *p* = 0.0111, respectively).

It is also important to note that a negative correlation was observed between the variance of tree-ring width (varTRW) and the level of individual heterozygosity. The correlation was weak and borderline significant according to the Pearson correlation coefficient (*r*_P_ = −0.115, *p* = 0.0805), but significant according to the Spearman correlation coefficient (*r*_S_ = −0.157, *p* = 0.0163), possibly indicating a homeostatic effect of increased heterozygosity. That is, trees with a higher level of heterozygosity had more stable development.

### 2.5. Associations Between Dendrophenotypes and Genotypes

According to the results of the General Linear Model (GLM), 15,223 SNPs were associated with at least one of the 24 dendrophenotypes (defol_1900, meanTRW, meanTRW30, Rc1, Rc2, Rc3, Rc4, Rc5, RRs1, RRs2, RRs4, RRs5, Rs1, Rs3, Rs4, Rs5, Rt1, Rt2, Rt3, Rt4, Rt5, rw_var_2006.2020, trend, varL, varTRW), including traits measured in all populations and traits measured in only one or a few populations. Among them, 12,719 SNPs were associated with one, 2216 SNPs with two, 272 SNPs with three, and 13 SNPs with four dendrophenotypic traits each (Table S2). Many SNPs were associated with the same dendrophenotypes in different drought periods (Table S2).

Among all SNPs found by GLM, 7940 SNPs were associated with one or more of the Rt, Rc, RR, and Rs indices measured for the five out of 15 drought periods (1937–1938, 1947, 1961, 1967, and 1988, respectively). Eight and three SNPs were associated with defoliation measured in 1900 and 1972 in the PS population, respectively, but none of them was the same for both periods. Associations were also found between needle size (meanL and varL) and 1304 SNPs. For five dendrophenotypes (meanTRW, meanTRW30, varTRW, varTRW2006-2020, and trend) reflecting tree ring widths, 6774 SNPs were found; 727 of them were associated with more than one trait. We did not annotate all of them, but only those 26 SNPs that were detected by at least one more method in addition to GLM. Their annotation is presented in Table S3 and described in Section 2.6.

According to the Bayesian Sparse Linear Mixed Model (BSLMM), 79 SNPs were associated with one of the 13 dendrophenotypes (Table S2). Half of them (41) were associated with one of the six defoliation traits (defol_1900, defol_1922, defol_1923, defol_1938, defol_1972, defol_1990) measured in the five periods, 1900, 1922, 1923, 1938, 1972, and 1990, respectively, in the PS population. In addition, 15 SNPs were associated in this population with variance of tree ring width measured for the 1976–1990, 1991–2005, and 2006–2020 periods.

Among 79 SNPs, 13 were associated with the Rc4, Rc9, Rs1, and Rt5 indices measured in the four drought periods (1937–1938, 1967, 1988, and 1990). Another 10 SNPs were associated with the mean and variance of tree ring width (meanTRW and varTRW), growth trend, and mean needle length (meanL and varTRW). None of 79 SNPs was associated with more than one dendrophenotype, and 24 of them that were detected by at least one more method in addition to BSLMM were further annotated and presented in detail in Table S3 and described in Section 2.6.

**Figure 6:**
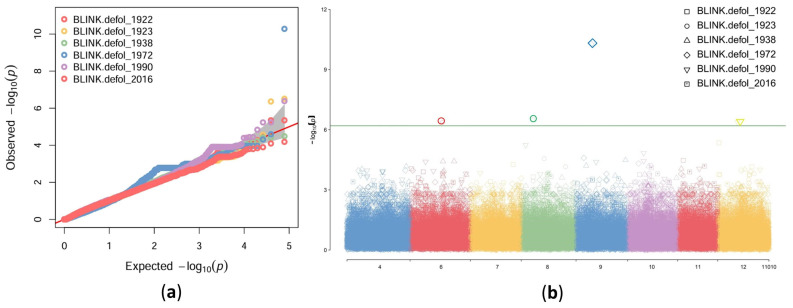
QQ (**a**) and Manhattan (**b**) plots presenting search results for SNPs associated with pine sawfly infestations in six different years: 1922, 1923, 1938, 1972, 1990, and 2016.

According to the Bayesian-information and Linkage-disequilibrium Iteratively Nested Keyway (BLINK), 40 SNPs were associated with one of 16 dendrophenotypes (defol_1923, defol_1972, defol_1990, mean_recov_p_dec, mean_resil_p_jun, meanL, meanTRW30, Rc4, RRs6, Rs11, Rs2, rw_var_1961.1975, rw_var_1976.1990, rw_var_1991.2005, rw_var_2006.2020, varTRW), including 11 SNPs associated with meanTRW measured in the periods 1961–1975, 1976–1990, 1991–2005 and 2006–2020 in the PS population and four SNPs associated with response to defoliation due to the pine sawfly outbreaks in 1923, 1972, and 1990 (Figure 6). None of 40 SNPs was associated with more than one dendrophenotype. All of them were further annotated and presented in detail in Tables S2 and S3 and described in Section 2.6.

**Figure 7:**
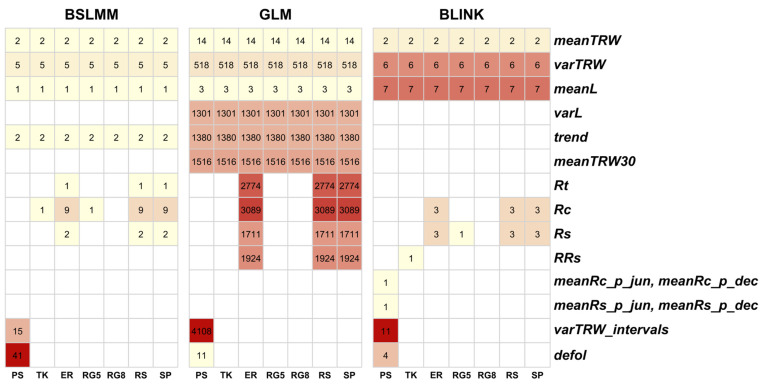
Heatmap of the GWAS results with numbers of SNPs in the cells detected by three methods, BSLMM, GLM, and BLINK. The color of cells is ranging from pale yellow to red, and its intensity reflects the number of SNPs associated with a dendrophenotype in the studied populations PS, TK, ER, RG5, RG*, RS, and SP (see Table 4 for their description). The number of SNPs associated with resistance (*Rt*), recovery (*Rc*), resilience (*Rs*), relative resilience (*RRs*), and defoliation (*defol*) indices is summed over all periods of stress. All dendrophenotypes and other traits are described in detail in Table 5. Dendrophenotypes with no associated SNPs are not presented in the graph.

In total, 15,310 SNPs presumably associated with dendrophenotypes were discovered by three different methods. They are presented in Figure 7.

**Figure 8:**
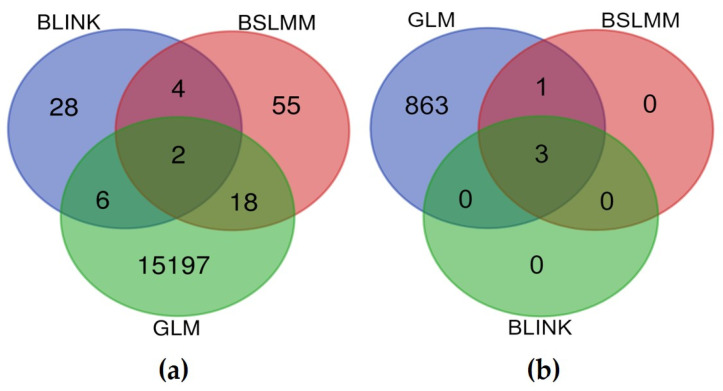
Venn diagram of SNP markers associated with dendrophenotypes (**a**) and mean values of dendrophenotypes *Rt*, *Rc*, *Rs*, and *RRs* (**b**) detected by three different methods—BLINK, BSLMM, and GLM.

Among them, 30 SNPs were detected by at least two different methods, and two SNPs by all three (Tables S2 and S3, Figure 8a). Among the two SNPs detected by all three methods, SNP 6.451121728 was associated with the mean needle length trait (meanL) measured in all populations and the relative resilience trait (RRs2) measured in the ER, RS, and SP populations, and SNP 12.656973718 was associated with the defoliation in 1990 trait (defol_1990) measured only in the PS population and with the needle length variance trait (varL) measured in all populations.

In addition, three SNPs associated with mean values of dendrophenotypes Rc) averaged across multiple years of climatic stresses in the TK, ER, RG, RS, and SP populations were also found by all three methods (Figure 8b).

No SNPs associated with Rt, Rc, Rs, and RRs were found for the following periods of drought stress: 1957, 1965, 1955, 1959, 2008, 1959–1961, 1965–1973, 1997. SNPs associated with at least one of the Rt, Rc, Rs, and RRs indices were found for the following periods of drought stress: 1937–1938, 1947, 1961, 1967, 1988, 1953, and 1990. No SNPs were found that were associated with the following dendrophenotypes: meanRt_p_jun, meanRt_p_dec, meanRt_p_MAR, meanRt_t_APR, meanRt_t_apr, meanRc_p_MAR, meanRc_t_apr, meanRs_p_MAR, meanRs_t_APR, meanRs_t_apr, meanRRs_p_jun, meanRRs_p_dec, meanRRs_p_MAR, meanRRs_t_APR, meanRRs_t_apr, prop_impact_defol, meanTRW_1931-1945, meanTRW_1946-1960, meanTRW_1961-1975, meanTRW_1976-1990, meanTRW_1991-2005, meanTRW_2006-2020, varTRW_1931-1945, varTRW_1946-1960, and defol_2016.

### 2.6. Annotation of SNPs

For 58 SNPs, including 30 SNPs detected by more than one association analysis method plus 28 SNPs detected by the BLINK method alone (considered as a method with the highest accuracy and reliability among the methods used), and three SNPs detected by all three methods and associated with mean Rc (61 SNPs in total), we analyzed the genomic regions in which these SNPs were localized (Table S3). Based on the genome annotation of a very closely related species, whitebark pine (*Pinus albicaulis* Engelm.), one SNP marker associated with defoliation in 1923 is located in chromosome 8 in the exonic part of the homolog of the gene encoding chromosome transmission fidelity protein. One of the two SNP markers associated with mean recovery dendrophenotype (Rc) is located in chromosome 7 in the 3′ region of the gene encoding uncharacterized protein, and another is located in chromosome 8 in the 5′ region of the gene encoding the SMR domain-containing protein At5g58720-like isoform X2. The remaining annotated markers with supposedly adaptive variation were located in intergenic regions at a distance of no closer than 1450 bp from the nearest gene (Table S3).

In total, among 61 SNPs associated with the variation of important adaptive traits and dendrophenotypes, 34 were located in the regions that contained annotated genes; three of them (CHR8.915883384, CHR10.1149421697, and CHR9.1029345389) were within the 10 Kbp regions of the genes encoding proteins (homologs of the heavy metal-associated isoprenylated plant protein 2, UV-stimulated scaffold protein A, and COBRA-like protein, respectively) and one (CHR8.397453659) within the exon of the gene encoding the chromosome transmission fidelity protein 18 homolog isoform X2 (Table S3). In addition, three SNPs, CHR7.1589497866, CHR8.460324735, and CHR8.397453659, were linked or associated with coding regions, but further than 10 Kbp from them. CHR7.1589497866, associated with the mean recovery dendrophenotype (Rc), is located in the 3′ region of the gene encoding an uncharacterized protein in chromosome 7 according to the available whitebark pine genome annotation. CHR8.460324735, also associated with the mean recovery dendrophenotype (Rc), is located in the 5′ region of the gene encoding the SMR domain-containing protein At5g58720-like isoform X2 in chromosome 8.

## 3. Discussion

The results of the presented analysis of genomic differentiation of Siberian stone pine populations are generally consistent with previous conclusions about the relatively weak population structure of large continuous conifer populations [17,18,19]. The obtained values of genetic differentiation between populations in our study are also consistent with the results of relatively low genetic differentiation between populations obtained earlier for Siberian stone pine based on allozyme and microsatellite markers [20,21,22,23]. Pairwise comparisons showed that the *F*_ST_ averaged 0.009, indicating a low degree of differentiation. Most of the genetic variance (98.3%) was within the studied populations, and only 1.7% of the genetic variance was due to differences between populations. PCA and population structure analyses also indicate relatively little genetic differentiation, as expected due to the relatively short distances between sampling sites. Only trees from the population on the territory of the Tomsk region (PS), located the most far from the other five populations, formed a separate cluster in the PCA with the largest level of differentiation observed between PS and TK (*F*_ST_ = 0.019). There was no correlation between geographic and genetic distances of the studied populations (Mantel-test *r* = 0.103, *p* = 0.313).

The search for associations between dendrophenotypes and individual heterozygosity revealed a correlation of heterozygosity with some dendrophenotypes. Heterosis was observed in most of these statistically significant cases, i.e., increased heterozygosity correlated with greater resistance to drought and defoliation. Signs of homeostasis were also observed, for example, increased heterozygosity correlated with a smaller variance in the TRW, i.e., trees with higher heterozygosity had more stable development and seemed less affected by environmental factors. A correlation between individual heterozygosity and some adaptive traits was also observed in studies of loblolly pine (*Pinus taeda* L.) [24], mountain hemlock (*Tsuga mertensiana* Bong. Carr) [25], European beech (*Fagus sylvatica* L.) [26] and Siberian larch (*Larix sibirica* Ledeb.) [5]. For a discussion of whether this is due to dominance (i.e., masking of unfavorable alleles and mutations in heterozygotes) or overdominance, see [27]. However, significant correlation values were obtained only for a few dendrophenotypes among 110 analyzed in our study in total, and only for a few phenotypes in the abovementioned studies. Moreover, the data are contradictory for some dendrophenotypes. It may indicate a rather complex association between individual heterozygosity and phenotypes.

The search for candidate genes with presumably adaptive variation correlated with variation in adaptive dendrophenotypes was carried out using three different methods and resulted in a data set of 61 SNPs. Annotation of genomic regions containing these SNPs showed that most of these markers are located in intergenic regions. Only three SNP markers, CHR7.1589497866, CHR8.460324735, and CHR8.397453659, were linked or associated with coding regions. CHR7.1589497866, associated with the mean recovery dendrophenotype (Rc), is located in the 3′ region of the gene encoding an uncharacterized protein in chromosome 7 according to the available whitebark pine genome annotation. CHR8.460324735, also associated with the mean recovery dendrophenotype (Rc), is located in the 5′ region of the gene encoding the SMR domain-containing protein At5g58720-like isoform X2 in chromosome 8. This protein is associated with various cellular functions, primarily related to DNA repair and maintenance. The SMR domain is typically found in proteins that are involved in DNA mismatch repair and other processes associated with DNA stability. Some SMR domain proteins are implicated in cell cycle regulation. They can influence cell cycle checkpoints, ensuring that cells do not proceed through the cycle with damaged DNA. These proteins are believed to be part of the plant’s response to DNA damage and environmental stress conditions (see [28] for review). CHR8.397453659, associated with defol_1923 (response to defoliation of 1923), is located in the exon of the homolog of the gene encoding chromosome transmission fidelity factor 18, which is a component of a replication factor C (RFC) complex that loads proliferating cell nuclear antigen (PCNA) on to DNA during the S phase of the cell cycle. The encoded protein may interact with other proteins, including RFC complex 3, to form a clamp loader complex that plays a role in sister chromatid cohesion during metaphase-anaphase transition.

Among 61 highly significant SNPs, the variation of 10 SNPs was associated with the response to defoliation by pine sawfly observed in the Tomsk pine forest near the village (PS population) in 1922, 1923, 1938, 1972, and 1990. Of these, six SNPs were associated also with the indices of resistance, recovery, and resilience calculated for the high mountain populations of the Western Sayan for the cold periods of 1947 and 1967.

## 4. Materials and Methods

### 4.1. Plant Material, Dendrochronology, and Dendrophenotypes

**Figure 9:**
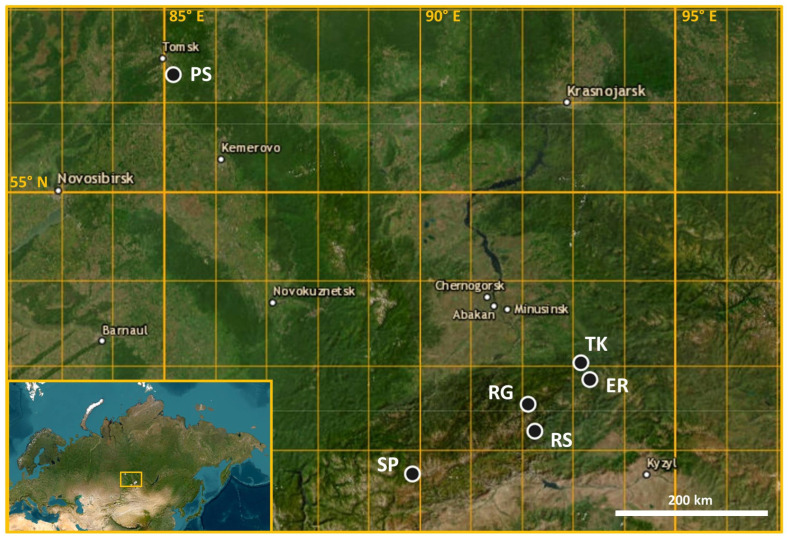
Geographic location of six Siberian stone pine populations in the study (PS, TK, ER, RG, RS, and SP).

Individual samples of needles for DNA extraction and wood samples (cores) for dendrophenotyping were collected in six populations of Siberian stone pine in Siberia: in the Tomsk region, Krasnoyarsk Krai, and the Republic of Khakassia (Table 4, Figure 9).

Wood cores of mature, intact living pine trees were taken at chest level. Samples were collected and processed using standard dendrochronological techniques [29]. Individual series of TRW measured using the LINTAB 5 device and the TSAP program v.4.67 [30] were cross-dated, and the dating was checked in the COFECHA program v.6.06p [31]. Using the ARSTAN program v.41d [32], age trends described by a 67% spline and the autocorrelation component were removed from the original series to highlight high-frequency fluctuations (including the climate signal). The generalized chronologies for sites were obtained by averaging with the binomial weighted mean. Dendroclimatic analysis of these generalized chronologies for Western Sayan was presented in [7,33]. Among these populations, those that are located in the lower part of the distribution range (RG and TK) are negatively affected by droughts and heat waves. Growth of populations located in the vicinity of the upper forest line (SP, RS, and ER) positively reacts to heat supply over the growth season, meaning growth depression occurs in the very cold and wet years.

To estimate reactions to recent climatic changes, mean values and linear TRW growth trends were calculated for standardized (after elimination of age trends) individual TRW series over 30 years prior to sampling, i.e., for period 1993–2022.

To analyze the effect of climatic (drought in PS, RG, and TK, but cold and humid spring-summer in SP, RS, and ER) and defoliation stresses on the growth of pine trees, the resistance (Rt = Gd/Gprev, where Gd is the average TRW during stress period, Gprev is the average TRW for 3 years before stress period), recovery (Rc = Gpost/Gd, where Gpost is the average TRW for 3 years after stress period), resilience (Rs = Gpost/Gprev) and relative resilience [RRs = (Gpost-Gd)/Gprev] indices proposed by Lloret et al. [34] were calculated and used as dendrophenotypes of individual tree responses to the stresses.

In all areas at the upper limit of Siberian stone pine growth, relatively synchronous dynamics of pine growth are observed, including synchronous suppression of growth throughout the Western Sayan caused by cold conditions of the growth season. The five reference stress periods of 1–3 years in length were selected there to calculate such dendrophenotypes as resistance (Rt), recovery (Rc), resilience (Rs), and relative resilience (RRs). In the foothills, the growth of pine is not synchronous between sites over large distances, probably due to lesser spatial synchronicity of precipitation as the main growth-limiting factor, so different reference years and longer periods of probably droughts and/or pest outbreaks were selected separately for each site. As a result, from 6 to 15 resistance indices were calculated for each population, taking into account their individual reference periods overlapping for some populations (Table 5).

While published data on the insect outbreaks in the studied Western Sayan area are lacking, the State Forest Committee of the Republic of Khakassia has unpublished archive data on the presence of dendrophagous insect outbreaks in Khakassian forests in 1999–2015. It can be assumed from these data that pine growth suppression by climatic extremes in the lower part of the distribution range in the Western Sayan could be exacerbated by pest damage.

In contrast, for the studied population in Tomsk Region (PS), repeated defoliation by the European pine sawfly *N. sertifer* is well documented in the archive of the Tomsk Forest Protection Center, which leads to a decrease in radial growth but does not cause the death of damaged trees [35]. However, data on defoliation are fragmentary and are available only since 1963. Therefore, to reconstruct defoliation, we used the method described in [36]. This method is implemented in the R package *dfoliatR* v.0.3.0 [37]. Scots pine *Pinus sylvestris* L. from a population located 5 km from PS was used as a non-defoliated species. The reconstruction results were in good agreement with the available archival data.

It should also be taken into account that in the valley of the Malaya Golaya River (RG population), specimens were collected from the trees growing at rather varying altitudes. Spatially and according to correlations between TRWs across these trees, this sample was divided into two subsamples, one at altitudes of 500–650 m above sea level (masl), and another at altitudes of 850–1000 masl. Separate chronologies were inferred for these subsamples that had different climatic responses and different years of low growth [33]. Correlations between genetic and geographic distance were also analyzed separately for these two subsamples.

In total, 110 different dendrophenotypes were used in the study, including Rt, Rc, Rs, and RRs indices for 15 reference periods of stress, and indices averaged over several reference periods, during which defoliation caused likely by pine sawfly was observed in the area near the village of Belousovo (PS), as well as the mean value and rate of increase in the TRW over the last 30 years before sampling (meanTRW and trend, respectively) and over the recent 30-year long period (meanTRW30), the average length of needles (meanL), and the variance of these traits (varTRW and varL). Tree age, geographic coordinates, and altitude were also calculated and used in this study (Table 5).

The radial growth of pine at the PS sampling site in the Tomsk region is influenced by temperature in April of both the previous and current year and precipitation in previous June and December and current March. We considered all those years when this influence (negative or positive) was significant for the entire sample. Correlation coefficients were calculated using the R package *treeclim* v.2.0.6.0 [38] using weather data from the Tomsk weather station located 19 km from the studied forest stand. To better take into account the relationship between the radial growth of pine and the weather, the Rt, Rc, Rs, and Rt indices were separately calculated for those years when the growth depended on the, e.g., April temperature of the current (meanRt_t_APR, meanRc_t_APR, meanRs_t_APR, meanRRs_t_APR) or the previous year (meanRt_t_apr, meanRc_t_apr, meanRs_t_apr, meanRRs_t_apr). To do this, we calculated the temperature quantile, below or above which the conditions for pine trees were considered stressful, and selected those years in which the temperature values were below or above the threshold value. For resilience indexes Rs, we chosen those values that were calculated for the years selected at the previous year, if the increase depended on the conditions of the current year (extension “APR” in the name of the dendrophenotype), or for the previous years (extension “apr”, respectively), if the dependence was on the conditions of the previous year. To reduce the number of parameters, averaged values were taken (numerous indices for each tree calculated for each year with cold April were averaged for Rt, Rc, Rs, and Rt).

The dependence on precipitation was taken into account similarly, although it was more complex (meanRt_p_MAR, meanRc_p_MAR, meanRs_p_MAR, meanRRs_p_MAR, meanRt_p_jun, meanRc_p_jun, meanRs_p_jun, meanRRs_p_jun, meanRt_p_dec, meanRc_p_dec, meanRs_p_dec, meanRRs_p_dec). The most obvious connection was between growth in the current year and precipitation in June of the previous season, i.e., the effect of moisture conditions on the accumulation of nutrients in moderately drained habitats appears in the next year. The negative effect of precipitation in December on growth in the following year can be interpreted as resulting from damage to the crown by heavy snowfall that can break or damage branches [39]. Finally, precipitation in March of the current year presumably affects the supply of moisture to trees during the period of spring reactivation. The positive effect of spring precipitation on radial growth has also been noted for other conifer species [40,41].

A decline in growth that lasted at least five years was considered a sign of defoliation (“defol”). We considered also synchronicity in manifestation of decline in growth among at least 25% of trees as a sign that allowed us to separate declines in growth caused by defoliation from other factors. It should also be taken into account that the maximum slowdown in growth is observed only 1–2 years after defoliation. Pine sawfly eats the most needles closer to autumn, but the next year radial growth can still be provided by previously stored nutrients. The maximum decline in growth was observed in 1922, 1923, 1938, 1972, 1990, and 2016 (defol_1922, defol_1923, defol_1938, defol_1972, defol_1990, and defol_2016, respectively).

### 4.2. DNA Extraction and ddRADseq

DNA was isolated from the needles of 243 Siberian pine trees using the CTAB method [42]. DNA concentration was assessed using a Qubit 2.0 fluorimeter (Thermo Fisher Scientific, Waltham, MA, USA), and the purity and quality of isolated DNA was assessed using an Implen NanoPhotometer P330 (Implen, München, Germany). High-quality DNA samples with A260/230 values of about 1.8 and a concentration of 20–150 ng/μL were selected for sequencing.

The preparation of ddRADseq libraries was based on a modified protocol from [43]. DNA samples were digested by two restriction enzymes, *EcoR*I and *Mse*I [44]. Paired-end sequencing of ddRADseq libraries was carried out in two independent runs on eight lanes of a NovaSeq 6000 sequencer (Illumina, San Diego, CA, USA) with 2 × 150 cycles and 28–30 samples per lane.

### 4.3. SNP Calling

The obtained sequencing data went through several stages of initial processing: filtering, trimming according to quality indicators, and demultiplexing using the *process_radtags* utility program included in the Stacks software v.2.5 [45]. Nucleotide reads from each sample were then aligned to a publicly available genome assembly of a closely related species, whitebark pine (*Pinus albicaulis*) [46] using Bowtie2 v.2.3 [47].

The search for SNPs was carried out using the *Gstacks* utility program included in the Stacks software v. 2.5. The resulting set of alignment-covered loci was subjected to several stages of filtering using the *Populations* utility program in such a way as to retain only loci that were represented in all studied populations by at least 80% of the loci in each tree and in 80% of the trees in each sample. The maximum level of observed heterozygosity at a nucleotide position was not allowed to exceed 0.7, and the frequency of the minor allele should not be less than 0.05. Imputation of missing genotypes was performed using the LD-kNNi method in TASSEL v.5.0 [48].

### 4.4. Genetic Structure of Populations

General measures of genetic diversity such as mean observed (*H_O_*) and expected (*H_E_*) heterozygosity, nucleotide diversity (π), mean indexes of fixation (*F*_IS_), and corresponding standard errors (SE) were calculated using Stacks v2.5. Pairwise *F*_ST_ values across all populations and their confidence intervals (based on 10,000 bootstrap samples) were estimated using the R package StAMPP v.1.6.3 [49]. To identify population structure from allele frequency data, PCA was performed using the R package ade4 v.1.7-22 [50]. To better understand population structure, we searched for the most likely number of clusters or subpopulations *K* for *K* from 1 to 15, with 20 replicates for each *K*, and estimated the contribution of each cluster to the genome of individual trees (*Q*-values) using the Admixture program v.1.3 implemented in the AdmixPipe pipeline v.3.0 [51] and the Δ*K* method [52].

Hierarchical analysis of molecular variance (AMOVA) was performed using Arlequin v.3.5.1.2 [53]. The correlation of pairwise geographic distances with genetic distances was calculated with the Mantel test implemented in the R-program *vegan* v.2.6 [54] using the matrixes of pairwise geographic distances (inferred from the averaged geographic coordinates) and the standard Nei’s genetic distances [55] (calculated from allele frequencies of SNP markers) between six populations.

### 4.5. Associations Between Dendrophenotypes and the Level of Individual Heterozygosity

For each tree, the individual level of heterozygosity was calculated as the number of heterozygous SNPs divided by the total number of SNPs genotyped for a given tree. Correlations between individual heterozygosity and dendrophenotypes were analyzed with Spearman’s rank correlation and Pearson’s correlation coefficients using the R package statistics v.3.6.2 [56].

### 4.6. Associations Between Dendrophenotypes and Genotypes

Genotype–dendrophenotype associations were analyzed using three approaches and corresponding models: General Linear Model (GLM), Bayesian Sparse Linear Mixed Model (BSLMM), and Bayesian-information and Linkage-disequilibrium Iteratively Nested Keyway (BLINK), respectively. For GLM, implemented in the TASSEL program [48], each site is an independent variable, and each dendrophenotype is a dependent (response) variable. SNPs were selected whose variability correlated with the variability of dendrophenotypes and which passed the FDR filtering threshold by an adjusted *p*-value < 0.05.

The SNP genotype–dendrophenotype associations were also assessed using BSLMM implemented in the GEMMA software package v.0.97 [57]. BSLMM is a polygenic model that accounts for the contribution of single SNPs with large effects as well as the simultaneous contribution of multiple SNPs with smaller effects in phenotypic variation. To achieve this, BLSMM includes the main effects of individual SNPs as predictors of the phenotype and the polygenic effect resulting from the combination of multiple SNPs with small effects. To identify significant genotype–phenotype associations for each trait, a posterior inclusion probability (*PIP*) threshold of >0.25 was used.

The third approach, BLINK [58], implemented in the R package GAPIT v.3.0 (Genome Association and Prediction Integrated Tool) [59], was used with default run parameters.

Since the samples for each population were too small to investigate associations within each population, the analysis was performed for all populations where a given trait was measured, except for those cases when a trait was analyzed only in one population. In particular, when choosing the reference periods of drought for calculating resistance indices, we used only those populations that showed synchronous growth dynamics and synchronous growth suppression. Therefore, for individual populations, the number of dendrophenotypes of resistance indices varied from 6 to 15 based on their individual reference periods. Dendrophenotypes associated with the response to defoliation were studied only in the population from Tomsk. Six dendrophenotypes, such as mean and variance of tree-ring width, mean and variance of needle length, and trend (meanTRW, varTRW, meanTRW30, meanL, varL, trend), were studied in all populations. The data on all traits and populations in which they were measured are presented in Table 5 and Table S2.

### 4.7. Annotation of SNPs

To analyze the genomic regions in which 30 SNPs were located that were detected by multiple association analysis methods, as well as for the 28 SNPs detected by the BLINK method and three SNPs associated with mean values of recovery dendrophenotype (Rc) averaged across multiple years of climatic stresses, we used the annotation of publicly available genome assembly of a closely related species, whitebark pine (*P. albicaulis*) [46]. Annotation of selected significant SNPs was carried out using the SNPdat program v.1.0.5 [60].

## 5. Conclusions

Among 61 SNPs associated with the variation of important adaptive traits and dendrophenotypes, 34 were located in the regions that contained annotated genes, three SNPs (CHR8.915883384, CHR10.1149421697, and CHR9.1029345389) within the 10 Kbp regions of the genes encoding proteins, and one (CHR8.397453659) within the exon of the gene encoding the chromosome transmission fidelity protein 18 homolog isoform X2 (Table S3). We certainly realize the limits of the RADseq method and that the obtained genomic data could be insufficient to uncover all the genes and genetic mechanisms that are responsible for adaptation to climatic extremes and defoliation, although those that are found could be of interest for the scientific community. We identified 61 SNPs whose variation was associated with important adaptive dendrophenotypes. Based on these results, it can be assumed that Siberian stone pine has relatively high adaptive genetic variation and potential in the studied area. It seems well adapted to abiotic stresses such as droughts. However, the negative growth trends observed for the recent decades in 6–49% of trees depending on population and more extensive in the low-elevation stands of the Western Sayan could be a sign of the beginning negative effect of climate change on the lower (and southern) fringes of Siberian stone pine forests. Therefore, in the highlands the trends are positive; that is, warming promotes the spread and growth of Siberian stone pine. Down the slope, the situation is less clear; there, the negative impact of droughts may appear. The results of this study will allow for a deeper understanding of the genetic mechanisms underlying the adaptations of Siberian stone pine to various climatic conditions and stresses. This study allowed us to detect important genes, and we obtained SNPs with significant adaptive variation that can be potentially used as genetic markers in marker-aided breeding for stress tolerance as well as for creating a SNP genotyping assay for monitoring adaptive genetic variation in other Siberian stone pine populations. However, more SNPs, preferably based on whole-genome sequencing, are required for developing a comprehensive high-density SNP genotyping assay to study Siberian stone pine. The presented approach, which includes both dendrophenotypic and genetic data and three different methods to find significant consensus SNPs, is novel and used for the first time in this species and can serve as a scientific basis for optimizing nature management, developing methods for the rational use of the studied species, identifying populations with good genetic potential, and conducting environmental monitoring.

## Figures and Tables

**Table 1:** Parameters of nucleotide diversity (π), observed (*H*_o_) and expected (*H*_e_) heterozygosity, and fixation index (*F*_IS_) of Siberian stone pines with standard error (SE).

Population	π	*H*_o_ ± SE	*H*_e_ ± SE	*F*_IS_ ± SE
PS	0.00085	0.204 ± 0.0005	0.204 ± 0.0005	0.016 ± 0.005
TK	0.00089	0.210 ± 0.0005	0.212 ± 0.0005	0.027 ± 0.003
ER	0.00091	0.220 ± 0.0005	0.222 ± 0.0004	0.017 ± 0.007
RG	0.00090	0.204 ± 0.0004	0.219 ± 0.0004	0.072 ± 0.008
RS	0.00087	0.198 ± 0.0004	0.212 ± 0.0004	0.065 ± 0.008
SP	0.00090	0.214 ± 0.0005	0.219 ± 0.0004	0.028 ± 0.008

**Table 2:** The genetic differentiation parameter (*F*_ST_) calculated between populations of Siberian stone pines with confidence intervals in square brackets.

Population	PS	TK	ER	RG	RS
**TK**	0.0188 [0.0184–0.0192]				
**ER**	0.0176 [0.0173–0.0180]	0.0052 [0.0049–0.0054]			
**RG**	0.0163 [0.0160–0.0166]	0.0031 [0.0029–0.0034]	0.0039 [0.0038–0.0040]		
**RS**	0.0181 [0.0179–0.0185]	0.0057 [0.0054–0.0060]	0.0050 [0.0048–0.0052]	0.0033 [0.0031–0.0034]	
**SP**	0.0150 [0.0146–0.0153]	0.0060 [0.0058–0.0063]	0.0054 [0.0052–0.0056]	0.0040 [0.0038–0.0041]	0.0041 [0.0039–0.0042]

**Table 3:** The AMOVA results and estimated Wright fixation indices (*F*-indices) for each level of the population hierarchy among six populations of Siberian stone pine.

Source of Variation	Sum of Squares	Variance Components	Percentage Variation	*F*-Indices
Among groups	22,275.6	115.2	1.2	*F*_ST_ = 0.017 *F*_SC_ = 0.005 *F*_CT_ = 0.012
Among populations within groups	53,233.7	47.9	0.5	
Within populations	4,333,517.1	9405.6	98.3	
Total	4,409,026.4	9568.7	100	

**Table 4:** Name, site, sample size, and geographic coordinates of six Siberian stone pine populations in the study.

Population	Location	Number of Trees	Elevation (masl)	Geographic Coordinates
PS	Near the village of Belousovo, 25 km southeast of the city of Tomsk	25	140–180	56.30969 N° 85.17264 E°
TK	Tensin Spring, Ergaki Nature Park, foothills of the Ergaki Mountain Range, Western Sayan Mountains, Krasnoyarsk Krai, Southern Siberia	18	420–460	53.039444 N° 93.155833 E°
ER	Lakes Uyutnoye and Raduzhnoye, Ergaki Nature Park, Ergaki Mountain Range, Western Sayan Mountains, Krasnoyarsk Krai, Southern Siberia	50	1400–1500	52.839667 N° 93.328000 E°
RG	Malaya Golaya River, Natural Biosphere Reserve “Sayano-Shushensky”, Western Sayan Mountains, Krasnoyarsk Krai, Southern Siberia	50	500–1000	52.55529462 N° 92.11411037 E°
RS	Sarla River, Natural Biosphere Reserve “Sayano-Shushensky”, Western Sayan Mountains, Krasnoyarsk Krai, Southern Siberia	50	1550–1700	52.23402773 N° 92.24586698 E°
SP	Sayan Pass, Western Sayan Mountains, Republic of Khakassia, Southern Siberia	50	2000–2050	51.7144165 N° 89.8624165 E°

**Table 5:** Description of dendrophenotypes and traits measured in the trees of Siberian stone pine in six populations and used in this study.

Dendrophenotypes and Traits	Description (Reference Years)	PS	TK	ER	RG5	RG8	RS	SP
meanTRW	mean tree-ring width over the entire observation period for all populations (1931–2020 for PS, 45–489 years for TK, ER, RG, RS, and SP)	+	+	+	+	+	+	+
varTRW	variance of tree-ring width over the entire observation period (1931–2020 for PS, 45–489 years for TK, ER, RG, RS, and SP)	+	+	+	+	+	+	+
meanL	mean needle length for first-year needles (2022)	+	+	+	+	+	+	+
varL	variance of needle length for first-year needles (2022)	+	+	+	+	+	+	+
trend	radial growth trends over the 30-year period prior to sampling for all populations (1993–2022)	+	+	+	+	+	+	+
meanTRW30	mean tree-ring width over the 30-year period prior to sampling	+	+	+	+	+	+	+
Rt1, Rt2, Rt3, Rt4, Rt5	resistance index (1937–1938, 1947, 1961, 1967, 1988)	-	-	+	-	-	+	+
Rt6, Rt7, Rt8	resistance index (1953, 1957, 1965)	-	+	-	-	-	-	-
Rt9	resistance index (1990)	-	+	-	+	-	-	-
Rt10, Rt11, Rt12	resistance index (1955, 1959, 2008)	-	-	-	+	-	-	-
Rt13, Rt14, Rt15	resistance index (1959–1961, 1965–1973, 1997)	-	-	-	-	+	-	-
Rc1, Rc2, Rc3, Rc4, Rc5	recovery index (1937–1938, 1947, 1961, 1967, 1988)	-	-	+	-	-	+	+
Rc6, Rc7, Rc8	recovery index (1953, 1957, 1965)	-	+	-	-	-	-	-
Rc9	recovery index (1990)	-	+	-	+	-	-	-
Rc10, Rc11, Rc12	recovery index (1955, 1959, 2008)	-	-	-	+	-	-	-
Rc13, Rc14, Rc15	recovery index (1959–1961, 1965–1973, 1997)	-	-	-	-	+	-	-
Rs1, Rs2, Rs3, Rs4, Rs5	resilience index (1937–1938, 1947, 1961, 1967, 1988)	-	-	+	-	-	+	+
Rs6, Rs7, Rs8	resilience index (1953, 1957, 1965)	-	+	-	-	-	-	-
Rs9	resilience index (1990)	-	+	-	+	-	-	-
Rs10, Rs11, Rs12	resilience index (1955, 1959, 2008)	-	-	-	+	-	-	-
Rs13, Rs14, Rs15	resilience index (1959–1961, 1965–1973, 1997)	-	-	-	-	+	-	-
RRs1, RRs2, RRs3, RRs4, RRs5	relative resilience index (1937–1938, 1947, 1961, 1967, 1988)	-	-	+	-	-	+	+
RRs6, RRs7, RRs8	relative resilience index (1953, 1957, 1965)	-	+	-	-	-	-	-
RRs9	relative resilience index (1990)	-	+	-	+	-	-	-
RRs10, RRs11, RRs12	relative resilience index (1955, 1959, 2008)	-	-	-	+	-	-	-
RRs13, RRs14, RRs15	relative resilience index (1959–1961, 1965–1973, 1997)	-	-	-	-	+	-	-
meanRt_p_jun, meanRt_p_dec	mean resistance index for years when the TRW depended on the precipitation in June or December of the previous year	+	-	-	-	-	-	-
meanRt_p_MAR	mean resistance index for years when the TRW depended on the precipitation in March of the current year	+	-	-	-	-	-	-
meanRt_t_APR	mean resistance index for years when the TRW depended on the temperature in April of the current year	+	-	-	-	-	-	-
meanRt_t_apr	mean resistance index for years when the TRW depended on the temperature in April of the previous year	+	-	-	-	-	-	-
meanRc_p_jun, meanRc_p_dec	mean recovery index for years when the TRW depended on the precipitation in June or December of the previous year	+	-	-	-	-	-	-
meanRc_p_MAR	mean recovery index for years when the TRW depended on the precipitation in March of the current year	+	-	-	-	-	-	-
meanRc_t_apr	mean recovery index for years when the TRW depended on the temperature in April of the previous year	+	-	-	-	-	-	-
meanRs_p_jun, meanRs_p_dec	mean resilience index for years when the TRW depended on the precipitation in June or December of the previous year	+	-	-	-	-	-	-
meanRs_p_MAR	mean resilience index for years when the TRW depended on the precipitation in March of the current year	+	-	-	-	-	-	-
meanRs_t_APR	mean resilience index for years when the TRW depended on the temperature in April of the current year	+	-	-	-	-	-	-
meanRs_t_apr	mean resilience index for years when the TRW depended on the temperature in April of the previous year	+	-	-	-	-	-	-
meanRRs_p_jun, meanRRs_p_dec	mean relative resilience index for years when the TRW depended on the precipitation in June or December of the previous year	+	-	-	-	-	-	-
meanRRs_p_MAR	mean relative resilience index for years when the TRW depended on the precipitation in March of the current year	+	-	-	-	-	-	-
meanRRs_t_APR	mean relative resilience index for years when the TRW depended on the temperature in April of the current year	+	-	-	-	-	-	-
meanRRs_t_apr	mean relative resilience index for years when the TRW depended on the temperature in April of the previous year	+	-	-	-	-	-	-
mean Rt, Rc, Rs, RRs	averaged across multiple years of climatic stresses	-	+	+	+	+	+	+
prop_impact_defol	proportion of trees that responded to the loss of needles	+	-	-	-	-	-	-
meanTRW_1931-1945, meanTRW_1946-1960, meanTRW_1961-1975, meanTRW_1976-1990, meanTRW_1991-2005, meanTRW_2006-2020	mean annual tree-ring width for the time periods 1931–1945, 1946–1960, 1961–1975, 1976–1990, 1991–2005, 2006–2020	+	-	-	-	-	-	-
varTRW_1931-1945, varTRW_1946-1960, varTRW_1961-1975, varTRW_1976-1990, varTRW_1991-2005, varTRW_2006-2020	variance of annual tree ring width for the time periods 1931–1945, 1946–1960, 1961–1975, 1976–1990, 1991–2005, 2006–2020	+	-	-	-	-	-	-
defol_1922, defol_1923, defol_1938, defol_1972, defol_1990, defol_2016	the presence of a fairly deep and sharp decline in growth caused by defoliation in 1922, 1923, 1938, 1972, 1990, 2016	+	-	-	-	-	-	-

“+”—measured, “-”—not measured.

## Data Availability

The data presented in this study are all available in the article and the files with the Supplementary Materials.

## Supplementary Materials

The following supporting information can be downloaded at: https://www.mdpi.com/article/10.3390/ijms252111767/s1.
