# Supplementary material for: Study of the Genetic Mechanisms of Siberian Stone Pine (Pinus sibirica Du Tour) Adaptation to the Climatic and Pest Outbreak Stresses Using Dendrogenomic Approach
Source: Int J Mol Sci. 2024 Nov 1;25(21):11767. doi: 10.3390/ijms252111767 (PMC11546098; doi:10.3390/ijms252111767)
Supplement: Supplementary file 1 [file ijms-25-11767-s001.zip › FigureS1_Contribution of each of the clusters (Q-values) for K = 2, 3, 4, 5, 12.pdf]

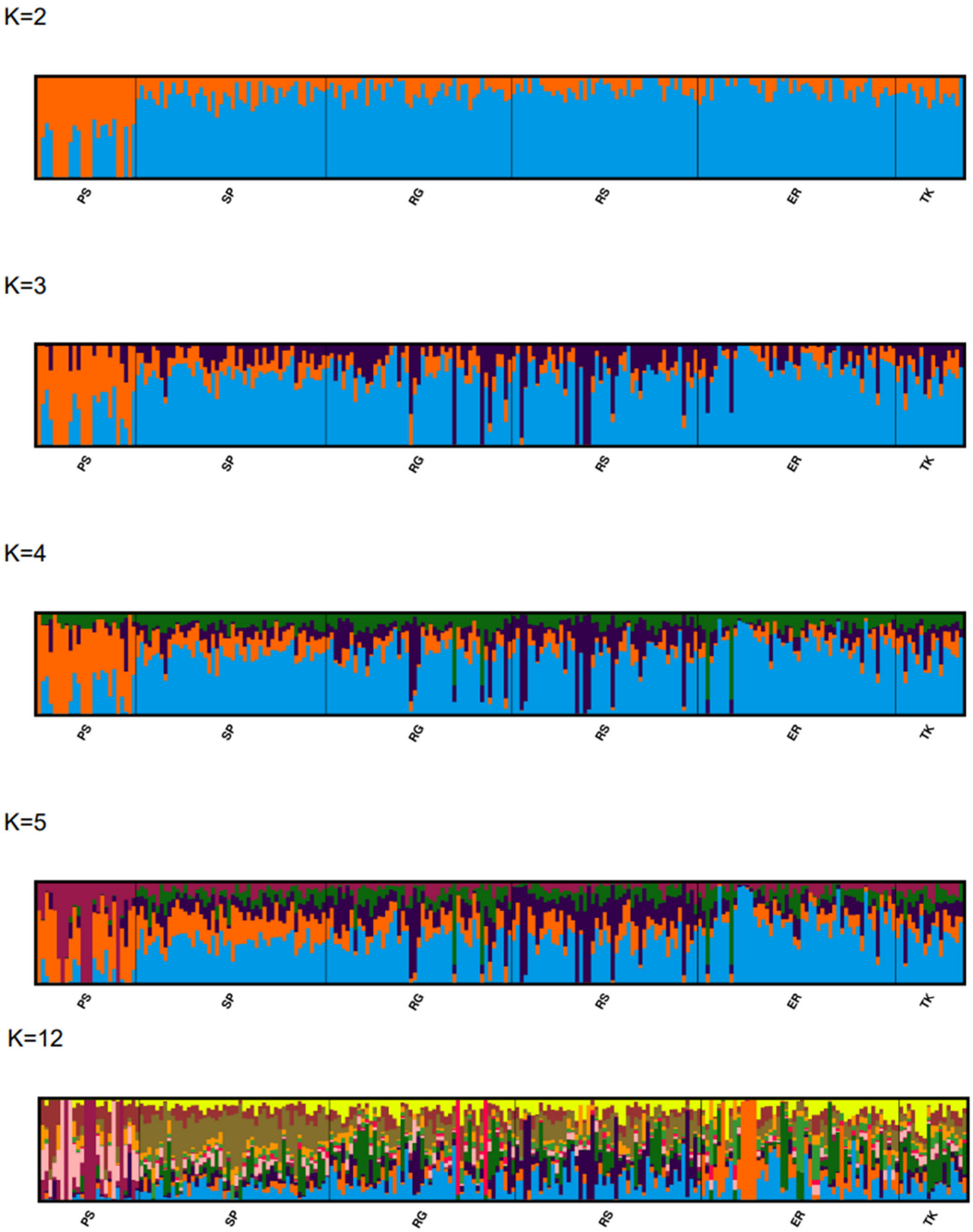

Figure S1. Contribution of each of the clusters (*Q*-values) for $K = 2, 3, 4, 5$, and 12 to individual Siberian pine trees from six populations (PS, TK, ER, RS, RG and SP).
