# Supplementary material for: Study of the Genetic Mechanisms of Siberian Stone Pine (Pinus sibirica Du Tour) Adaptation to the Climatic and Pest Outbreak Stresses Using Dendrogenomic Approach
Source: Int J Mol Sci. 2024 Nov 1;25(21):11767. doi: 10.3390/ijms252111767 (PMC11546098; doi:10.3390/ijms252111767)
Supplement: Supplementary file 1 [file ijms-25-11767-s001.zip › Table S1_Pearson and Spearman corr coeff between the level of ind heterozygosity and dendrophenotypes.pdf]

Table S1. Values of Pearson and Spearman correlation coefficients between the level of individual heterozygosity and dendrophenotypes.

**Dendrophenotype**	**Pearson correlation coefficient**						**Spearman's rank correlation coefficient**		
	t	**d.f.**	r_P	***ll***	***ul***	***P***	***S***	r_S	***P***
**Rt1**	**-2.118**	123	**-0.188**	-0.352	-0.012	**0.0362**	297404	0.086	0.3380
**Rt2**	**2.556**	132	**0.217**	0.049	0.373	**0.0117**	286674	**0.285**	**0.0009**
**Rt3**	**2.146**	137	**0.180**	0.014	0.337	**0.0337**	338978	**0.243**	**0.0041**
**Rt13**	**3.905**	25	**0.616**	0.307	0.807	**0.0006**	1628	**0.503**	**0.0082**
**Rs1**	**-2.350**	123	**-0.207**	-0.369	-0.033	**0.0204**	328396	-0.009	0.9215
**Rs2**	**2.525**	132	**0.215**	0.047	0.371	**0.0128**	284324	**0.291**	**0.0007**
**Rs13**	**2.690**	25	**0.474**	0.114	0.723	**0.0126**	1978	**0.396**	**0.0417**
**meanL**	**-2.482**	232	**-0.161**	-0.283	-0.033	**0.0137**	2430708	**-0.138**	**0.0345**
**defol_1972**	**2.376**	23	**0.444**	0.059	0.714	**0.0262**	1047	**0.597**	**0.0016**
**trend**	**2.070**	205	**0.143**	0.007	0.274	**0.0402**	1388336	0.061	0.3839
**meanTRW30**	**-2.008**	205	**-0.139**	-0.270	-0.003	**0.0460**	1738758	**-0.176**	**0.0111**
**varTRW**	-1.756	231	-0.115	-0.240	0.014	0.0805	2439602	**-0.157**	**0.0163**
**RRs3**	-1.754	137	-0.148	-0.307	0.019	0.0817	543404	**-0.214**	**0.0115**
Rt14	-1.759	25	-0.332	-0.632	0.055	0.0909	3924	-0.198	0.3212
**Rc3**	-1.663	137	-0.141	-0.300	0.026	0.0985	548946	**-0.226**	**0.0075**
RRs11	1.739	18	0.379	-0.076	0.704	0.0991	1012	0.239	0.3086
Rt5	-1.654	141	-0.138	-0.295	0.027	0.1003	515016	-0.057	0.5001
Rs5	-1.643	141	-0.137	-0.295	0.028	0.1025	516658	-0.060	0.4750
mean_recov_p_dec	1.626	23	0.321	-0.085	0.636	0.1177	1692	0.349	0.0876
Rs6	-1.654	13	-0.417	-0.766	0.121	0.1220	724	-0.293	0.2887
Rc11	1.614	18	0.356	-0.103	0.690	0.1239	1134	0.147	0.5338
Rs15	-1.549	25	-0.296	-0.608	0.095	0.1338	3800	-0.160	0.4238
defol_1923	1.440	21	0.300	-0.128	0.634	0.1647	1480	0.269	0.2152
RRs1	-1.395	123	-0.125	-0.294	0.052	0.1655	348056	-0.069	0.4420
mean_resil_p_dec	1.389	23	0.278	-0.131	0.607	0.1782	1662	0.361	0.0771
Rc15	-1.362	25	-0.263	-0.584	0.130	0.1855	3670	-0.120	0.5486
Rs14	-1.360	25	-0.263	-0.584	0.130	0.1858	4416	-0.348	0.0759
RRs15	-1.308	25	-0.253	-0.578	0.140	0.2027	3598	-0.098	0.6245
prop_impact_defol	1.272	23	0.256	-0.154	0.592	0.2160	1620	0.377	0.0632
Rc4	1.145	137	0.097	-0.070	0.260	0.2542	434608	0.029	0.7345
mean_resist_t_APR	-1.166	23	-0.236	-0.577	0.175	0.2556	3260	-0.254	0.2199
mean_rel,resil_p_dec	1.125	23	0.228	-0.183	0.572	0.2721	1612	0.380	0.0618
varL	1.080	232	0.071	-0.058	0.197	0.2811	2158159	-0.011	0.8714
elev	-1.082	92	-0.112	-0.308	0.093	0.2821	170969	**-0.235**	**0.0225**
mean_resist_p_dec	1.099	23	0.223	-0.188	0.568	0.2830	1770	0.319	0.1200
mean_recov_p_MAR	1.088	23	0.221	-0.191	0.567	0.2881	1932	0.257	0.2142
lat	1.002	92	0.104	-0.101	0.300	0.3192	102608	**0.259**	**0.0118**
rw_mean_2006.2020	-1.011	23	-0.206	-0.556	0.206	0.3226	2420	0.069	0.7418
RRs4	0.990	137	0.084	-0.083	0.247	0.3240	435998	0.026	0.7621
Rc14	-0.974	25	0.191	-0.533	0.204	0.3392	4032	-0.231	0.2457

Dendrophenotype	Pearson correlation coefficient						Spearman's rank correlation coefficient		
	t	**d.f.**	r_P	***ll***	***ul***	***P***	***S***	r_S	***P***
Rt11	-0.927	18	-0.214	-0.599	0.253	0.3660	1154	0.132	0.5768
Rs11	0.914	18	0.211	-0.256	0.597	0.3728	878	0.340	0.1428
Rt4	-0.892	137	-0.076	-0.239	0.092	0.3739	484818	-0.083	0.3298
RRs7	0.918	13	0.247	-0.304	0.674	0.3751	438	0.218	0.4343
Rs12	0.879	18	0.203	-0.263	0.592	0.3911	1052	0.209	0.3748
Rc7	0.855	13	0.231	-0.319	0.664	0.4081	444	0.207	0.4578
defol_1922	-0.842	21	-0.181	-0.552	0.250	0.4093	2476	-0.223	0.3063
defol_1900	0.822	11	0.241	-0.358	0.699	0.4285	251	0.312	0.2997
mean_resil_p_MAR	0.797	23	0.164	-0.247	0.525	0.4338	2048	0.212	0.3068
RRs14	-0.786	25	-0.155	-0.505	0.239	0.4392	4124	-0.259	0.1916
Rs8	0.798	13	0.216	-0.333	0.656	0.4394	498	0.111	0.6953
mean_rel,resil_p_MAR	0.775	23	0.160	-0.251	0.522	0.4461	1988	0.235	0.2562
lon	-0.744	92	-0.077	-0.276	0.127	0.4587	158509	-0.145	0.1627
mean_recov_t_APR	0.741	23	0.153	-0.258	0.517	0.4660	2258	0.132	0.5292
meanTRW	-0.718	231	-0.047	-0.175	0.082	0.4734	2151377	-0.020	0.7557
Rt6	-0.728	13	-0.198	-0.645	0.350	0.4793	616	-0.100	0.7241
Rt10	-0.715	18	-0.166	-0.567	0.298	0.4840	1558	-0.171	0.4682
mean_resist_p_jun	0.709	23	0.146	-0.264	0.512	0.4856	2230	0.142	0.4957
Rs9	-0.691	35	-0.116	-0.424	0.216	0.4940	10152	-0.203	0.2264
rw_mean_1931.1945	-0.683	23	-0.141	-0.508	0.269	0.5017	2976	-0.145	0.4887
Rc13	0.669	25	0.133	-0.261	0.488	0.5097	2970	0.093	0.6419
mean_rel,resil_t_apr	0.665	23	0.137	-0.273	0.505	0.5129	2620	-0.008	0.9721
rw_var_1931.1945	0.657	23	0.136	-0.274	0.504	0.5179	2132	0.180	0.3875
mean_resist_p_MAR	0.615	23	0.127	-0.282	0.497	0.5444	2266	0.128	0.5390
rw_mean_1991.2005	-0.574	23	-0.119	-0.491	0.290	0.5719	2464	0.052	0.8038
defol_1938	0.573	23	0.119	-0.290	0.491	0.5722	1990	0.235	0.2588
RRs6	-0.557	13	-0.153	-0.617	0.390	0.5873	588	-0.050	0.8626
RRs12	0.541	18	0.126	-0.335	0.539	0.5955	1290	0.030	0.9013
Rs7	0.538	13	0.148	-0.394	0.614	0.5994	568	-0.014	0.9642
RRs13	0.527	25	0.105	-0.287	0.466	0.6027	3046	0.070	0.7272
rw_var_1961.1975	0.524	23	0.109	-0.299	0.483	0.6053	2290	0.119	0.5688
RRs2	0.512	132	0.044	-0.126	0.212	0.6097	376968	0.060	0.4911
mean_resil_t_apr	0.517	23	0.107	-0.301	0.482	0.6104	2590	0.004	0.9868
Rc12	0.504	18	0.118	-0.342	0.533	0.6204	1286	0.033	0.8913
mean_resil_t_APR	-0.494	23	-0.103	-0.478	0.305	0.6257	3278	-0.261	0.2072
Rc2	0.486	132	0.042	-0.128	0.210	0.6276	387164	0.034	0.6920
Rt8	0.484	13	0.133	-0.407	0.604	0.6366	406	0.275	0.3203
RRs10	0.466	18	0.109	-0.350	0.526	0.6467	1132	0.149	0.5296
mean_rel,resil_t_APR	0.455	23	0.094	-0.312	0.472	0.6535	2458	0.055	0.7953
rw_var_1946.1960	-0.452	23	-0.094	-0.471	0.313	0.6557	2522	0.030	0.8874
rw_var_2006.2020	0.427	23	0.089	-0.318	0.467	0.6737	2282	0.122	0.5599
mean_resil_p_jun	0.393	23	0.082	-0.324	0.462	0.6983	2510	0.035	0.8699
Rt7	-0.383	13	-0.106	-0.586	0.430	0.7080	700	-0.250	0.3677

Dendrophenotype	Pearson correlation coefficient						Spearman's rank correlation coefficient		
	t	d.f.	r_P	*ll*	*ul*	*P*	*S*	r_S	*P*
RRs9	-0.363	35	-0.061	-0.378	0.268	0.7188	9630	-0.142	0.4019
rw_mean_1946.1960	-0.346	23	-0.072	-0.454	0.333	0.7323	2653	-0.020	0.9229
Rt9	-0.323	35	-0.054	-0.372	0.274	0.7488	8778	-0.041	0.8113
defol_1990	-0.323	23	-0.067	-0.450	0.337	0.7496	2280	0.123	0.5583
defol_2016	0.306	23	0.064	-0.340	0.447	0.7626	2542	0.022	0.9161
RRs5	-0.302	141	-0.025	-0.189	0.139	0.7629	504986	-0.036	0.6674
Rt15	-0.300	25	-0.060	-0.430	0.328	0.7666	3174	0.031	0.8778
RRs8	0.265	13	0.073	-0.456	0.564	0.7949	512	0.086	0.7630
Rc1	-0.247	123	-0.022	-0.197	0.154	0.8054	343716	-0.056	0.5348
Rt12	0.231	18	0.054	-0.398	0.485	0.8197	1134	0.147	0.5338
rw_mean_1976.1990	0.230	23	0.048	-0.354	0.435	0.8201	2566	0.013	0.9516
mean_resist_t_apr	-0.211	23	-0.044	-0.432	0.357	0.8347	2484	0.045	0.8324
mean_recov_t_apr	0.204	23	0.042	-0.359	0.430	0.8405	2776	-0.068	0.7473
rw_mean_1961.1975	-0.203	23	-0.042	-0.430	0.359	0.8411	2549	0.020	0.9258
Rc10	0.189	18	0.045	-0.406	0.478	0.8519	1186	0.108	0.6488
Rc9	0.183	35	0.031	-0.296	0.351	0.8559	8858	-0.050	0.7681
Rc8	0.184	13	0.051	-0.474	0.549	0.8566	558	0.004	0.9949
Rs4	0.176	137	0.015	-0.152	0.181	0.8604	472002	-0.055	0.5230
Rc6	-0.173	13	-0.048	-0.547	0.476	0.8652	578	-0.032	0.9132
Rs3	-0.148	137	-0.013	-0.179	0.154	0.8823	474524	-0.060	0.4810
rw_var_1991.2005	0.054	23	0.011	-0.386	0.405	0.9573	2390	0.081	0.7010
mean_rel,resil_p_jun	-0.051	23	-0.011	-0.404	0.386	0.9598	2860	-0.100	0.6332
rw_var_1976.1990	-0.045	23	-0.009	-0.403	0.387	0.9645	2705	-0.040	0.8479
age	0.037	222	0.003	-0.129	0.134	0.9702	1866378	0.004	0.9568
Rc5	0.036	140	0.003	-0.162	0.168	0.9713	491094	-0.029	0.7304
mean_recov_p_jun	0.002	23	0.000	-0.395	0.396	0.9981	2742	-0.055	0.7953
Rs10	0.002	18	0.000	-0.442	0.443	0.9987	1162	0.126	0.5945

t - Student's t-test, *ll* and *ul* - lower and upper limits of confidence interval, r_P -Pearson correlation coefficient, *S* - the sum of all squared rank differences, r_S - Spearman's correlation coefficient. Significant values are highlighted by bold.
